# Genetic diversity and population structure of naturally rare *Calibrachoa* species with small distribution in southern Brazil

**DOI:** 10.1590/1678-4685-GMB-2017-0314

**Published:** 2019-03-11

**Authors:** Ana Laura de Wallau John, Geraldo Mäder, Jeferson N. Fregonezi, Loreta B. Freitas

**Affiliations:** 1 Universidade Federal do Rio Grande do Sul Universidade Federal do Rio Grande do Sul Department of Genetics Laboratory of Molecular Evolution Porto AlegreRS Brazil Laboratory of Molecular Evolution, Department of Genetics, Universidade Federal do Rio Grande do Sul, Porto Alegre, RS, Brazil

**Keywords:** Plastid DNA, microsatellites, threatened species, grasslands, herbaceous species

## Abstract

*Calibrachoa* is a South-American genus comprising 27 species, several considered endemic or rare; few were subjects in genetic studies. We attempted to generate new data about the phylogenetically related and rare species *C*. *eglandulata*, C. *sendtneriana*, *C*. *serrulata*, and *C*. *spathulata* concerning their genetic diversity and population structure, which, coupled with their known restricted distribution, could help access their conservation status and contribute to the study of the Brazilian biodiversity. We sequenced 88 individuals for plastid intergenic spacers and genotyped 186 individuals for five microsatellite loci. Compared among each other, *C. sendtneriana* and *C*. *serrulata* presented the highest values of genetic diversity [π% (sd) = 0.23 (0.14) and 0.43 (0.25), respectively], followed by *C*. *spathulata* [π% (sd) = 0.19 (0.12)] and *C*. *eglandulata* [π% (sd) = 0.02 (0.03)]. Population differentiation was evident for these latter species, whereas it was not significant for *C*. *sendtneriana* and *C*. *serrulata*. Factors such as habitat specificity and fragmentation, pollination syndrome, and life history could explain the observed patterns. Based on the new genetic data and the species’ biology, a conservation status was assigned for *C*. *sendtneriana* and the status of the other three species was reviewed.

## Introduction

In elevations from 800 to 1,800 m in southern Brazil, the vegetation forms a mosaic of *Araucaria* forest and grasslands known as the Brazilian subtropical highland grassland (BSHG) or Campos de Cima da Serra (Iganci *et al.*, 2011). These natural grasslands occur along the Serra Geral formation (Behling, 2002) in Rio Grande do Sul, Santa Catarina (SC), and Paraná (PR) states, and they harbor high levels of plant diversity and endemism (Iganci *et al.*, 2011). Currently, the advance of monocultures, forestry, and urbanization are the most important threats to the region (Behling *et al.*, 2005).

Endemic species are plants that occur in specific habitats and are geographically restricted, but such plants can be sparse or abundant, meaning that they can have different population sizes and spatial arrangements (Rabinowitz, 1981). A common assumption is that endemic species are rare and genetically depleted, presenting low genetic diversity and strong population differentiation (Binks *et al.*, 2015; Ciéslak *et al.*, 2015). However, many examples can be found in the literature that contradict this statement (Hou and Lou, 2011; Ciéslak *et al.*, 2015; Turchetto *et al.*, 2016). Absent or limited gene flow and inbreeding are putative drivers for the genetic diversity pattern associated with small or restricted population sizes (Ellstrand and Elam, 1993), and several other factors can influence the genetic diversity and population structure of rare species. These include mating system, pollination and seed dispersal vectors, life cycle, habitat specificity, demographic history, and landscape and/or climate changes (Loveless and Hamrick, 1984; Shirk *et al.*, 2014; Ciéslak *et al.*, 2015; Shao *et al.*, 2015).

As genetic diversity is directly linked to species survival via the ability to adapt to environmental changes (Ellstrand and Elam, 1993; Binks *et al.*, 2015), analysis of a species’ gene pool is fundamental to identify populations that have high and representative levels of genetic polymorphism. Such knowledge can guide strategies regarding how the population should be managed and how this management should be implemented (Casacci *et al.*, 2014; Ciéslak *et al.*, 2015).

*Calibrachoa* Cerv. (Solanaceae) is a South American genus of perennial herbs and small shrubs distributed in Argentina, Brazil, Paraguay, and Uruguay, with one species sporadically occurring in North America and Europe (Greppi *et al.*, 2013). The majority of the 27 species assigned to the genus occur in Brazil (Stehmann and Greppi, 2011), where several are endemic and at least five can be considered rare (Fregonezi *et al.*, 2013). *Calibrachoa* species inhabit open areas, grasslands, and rocky outcrops in subtropical and temperate regions. They have a barochoric seed dispersal system and are self-incompatible except for one species (Tsukamoto *et al.*, 2002; Fregonezi *et al.*, 2013).

**Figure 1:**
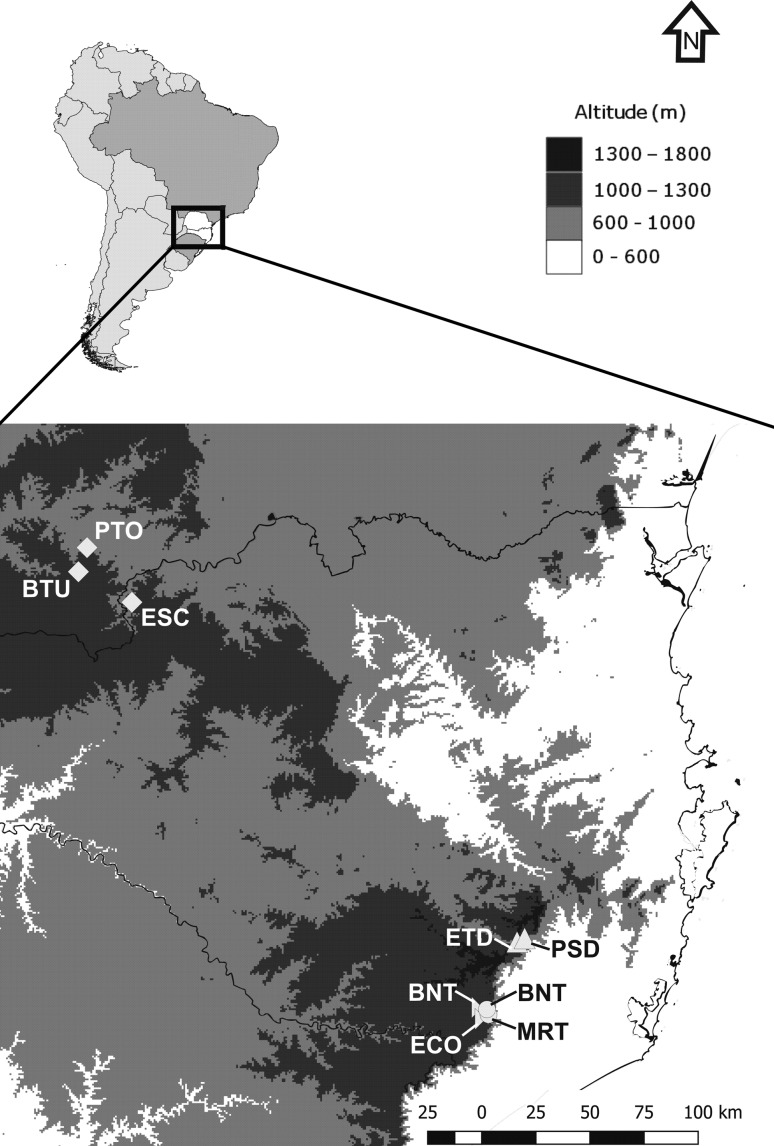
Geographic distribution of collection locations of four *Calibrachoa* species: *C. eglandulata* (triangles); *C. sendtneriana* (half-diamonds); *C. serrulata* (circles); and *C. spathulata* (diamonds). For population codes per species see Table 1.

The molecular phylogenetic analysis of the genus revealed two main clades corresponding to two subgenera (Fregonezi *et al.*, 2012). In the subgenus *Stimomphis*, four rare and related species occur exclusively in the highland grasslands in southern Brazil (Figure 1): *Calibrachoa eglandulata*, *C. sendtneriana*, *C. serrulata*, and *C. spathulata*.

**Table 1:** Sampling information for four *Calibrachoa* species.

Species	Population code	Geographic Coordinates	Vouchers^*^	Sample size (cpDNA)^#^	Sample size (SSR)^#^
*C. eglandulata*				16	54
	ETD	28 02’ 35’’S 49 24’ 30’’W	BHCB104869	9	40
	PSD	28 01’ 32’’S 49 22’ 21’’W	BHCB104877	7	14
*C. sendtneriana*				37	54
	BNT	28 21’ 14’’S 49 34’ 25’’W	BHCB116972	31	31
	ECO	28 24’ 31’’S 49 33’ 27’’W	ICN184926	6	23
*C. serrulata*				18	30
	BNT	28 21’ 46’’S 49 33’ 05’’W	ICN184945	8	10
	MRT	28 23’ 16’’S 49 32’ 38’’W	ICN184944	10	20
*C. spathulata*				17	48
	ESC	26 25’ 53’’S 51 14’ 11’’W	ICN160333	6	27
	BTU	26 17’ 12’’S 51 29’ 24’’W	ICN184918	6	12
	PTO	26 10’ 19’’S 51 26’ 57’’W	ICN184920	5	9

* BHCB – Herbarium of Universidade Federal de Minas Gerais, Belo Horizonte, MG, Brazil; ICN – Herbarium of Universidade Federal do Rio Grande do Sul, Porto Alegre, RS, Brazil.

# total number of analyzed individuals per species and per population.

Calibrachoa eglandulata (Figure S1) is a bee-pollinated species with pink flowers (Stehmann and Semir, 1997) that grows in rocky shaded environments and is federally listed as Endangered (Martinelli and Moraes, 2013); the only known occurrence sites for this species are in Urubici municipality, Santa Catarina (SC), Brazil. *Calibrachoa sendtneriana* (Figure S2) has orange-red flowers, suggesting a bird pollination syndrome (Fregonezi *et al.*, 2013) and currently has no determined conservation status. This species is a small shrub growing in the margins of cloud forests and in rocky outcrops in the middle of grasslands, recorded only in the municipalities of Bom Jardim da Serra and Bom Retiro (SC). *Calibrachoa serrulata* (Figure S3) is also bird-pollinated (Fregonezi *et al.*, 2013) even though it has magenta-colored flowers, and it grows in cliff protrusions along the hills; currently this species is ranked as Data Deficient (Martinelli and Moraes, 2013). There are records of its occurrence in Bom Jardim da Serra and Lauro Müller municipalities (SC). *Calibrachoa spathulata* (Figure S4) has magenta to purplish bee-pollinated flowers and it grows on roadsides close to urban areas in five cities located in the states of Paraná and Santa Catarina. It is considered a Vulnerable species (Martinelli and Moraes, 2013).

All these four *Calibrachoa* species are known to grow in very few localities and occupy small distribution areas; as it is common among *Calibrachoa* species, there are no specific studies on pollination or mating systems for these species. Only *C*. *serrulata* is not directly affected by anthropogenic pressure, because normally the individuals grow directly on vertical walls of canyons. These species can be considered rare based on their population size and range, and are currently threatened by habitat loss caused by human intervention.

Here, we aimed to estimate the genetic diversity and population structure of these four species and to suggest a new or a reviewed conservation status for each species through the analysis of new and important information. With only three studies published discussing genetic information for *Calibrachoa* species (Fregonezi *et al.*, 2012, 2013; Mäder *et al.*, 2013), this current study increases the scientific knowledge for this genus and the Brazilian flora, providing useful data for the preservation of these four species.

## Materials and Methods

### Sampling and DNA extraction

For each of the four *Calibrachoa* species (*C. eglandulata, C. sendtneriana, C. serrulata,* and *C. spathulata*), we covered the entire known geographic distribution, visiting all recorded collection sites. For *C*. *sendtneriana*, no plants were found in the site previously prospected (J.N. Fregonezi, personal observation), resulting in two or three sample collection sites (hereafter called populations) per species (Figure 1, Table 1). Populations of *C*. *eglandulata* (ETD and PSD) were located 4 km apart from each other, whereas 3.2 km separated *C*. *serrulata*‘s populations (BNT and MRT). The *C*. *sendtneriana*‘s BNT and ECO populations were 7.3 km away from each other. The populations of *C. spathulata* were the most distant from each other: ESC *vs.* BTU = 31.4 km, ESC *vs.* PTO = 37.6 km, and BTU *vs.* PTO = 13.4 km.

Young leaves were collected from each sampled individual, stored in silica gel, and pulverized using liquid nitrogen. DNA extraction was performed following the CTAB (cetyl-trimethylammonium bromide)-based method as described for *Calibrachoa* species (Fregonezi *et al.*, 2012). Genomic DNA quality was evaluated by horizontal electrophoresis in a 1% agarose gel stained with 0.001% GelRed (Biotium, Fremont, CA, EUA) and visualized under ultraviolet light. Concentration and purity were evaluated using a NanoDrop 1000 spectrophotometer (Thermo Fisher Scientific Inc., Waltham, MA, USA) by measuring the absorbance at 260 and 280 nm.

### Plastid markers

The plastid intergenic spacers *trnH*-*psbA* (Sang *et al.*, 1997) and *trnS*-*trnG* (Hamilton, 1999) were amplified for 88 individuals (Table 1) of four *Calibrachoa* species. PCR assays were performed using 0.2 mM of each dNTP, 0.16 mM of each primer, 2 mM MgCl_2_, 5 ng of template genomic DNA, 1 U Platinum Taq polymerase (Thermo-Fisher Scientific Co., Waltham, MA, USA), and 1 reaction buffer (Thermo-Fisher) for a total volume of 25 μL. Reaction conditions were as follows: 94 °C for 5 min, 35 cycles of 94 °C for 1 min, 55 °C for 1 min and 72 °C for 1.5 min, finalizing with 72 °C for 10 min. Sequences obtained in this study were deposited in GenBank (see Table S1 for accession numbers). After amplification, the quality of PCR products was checked by horizontal electrophoresis in a 1% agarose gel stained with 0.001% GelRed^TM^ (Biotium, Freemont, CA, USA) and later purified using PEG 20% (polyethylene glycol; Sigma-Aldrich Co., St. Louis, MO, USA) according to Dunn and Blattner (1987) and sequenced in a MegaBACE1000 (GE Healthcare Bio Sciences Corp., Piscataway, NY, USA) automatic sequencer according to the manufacturer’s instructions and the DYEnamicET Terminator Sequencing Premix Kit (GE Healthcare). For each marker, both forward and reverse strands were checked using the Chromas 2.0 software (Technelysium, Helensvale, Australia), manually aligned and edited using MEGA 6 (Tamura *et al.*, 2013); insertion/deletions events longer than one base pair (bp) were coded as single mutational events.

### Nuclear markers

For all these four species, we initially tested 25 microsatellite loci developed for other *Calibrachoa* species that presented positive transferability (Silva-Arias *et al.*, 2015; G. Mäder *et al.,* unpublished data). Because only some few of them could be amplified and were polymorphic for all the four species, we used four loci described for *C. heterophylla* (Che 18, Che 46, Che 59, and Che 34; Silva-Arias *et al.*, 2015) and one for *C. pygmaea* (Cpy58; G. Mäder *et al.,* unpublished data) to amplify the 186 individuals of the four *Calibrachoa* species (Table 1). PCR final volume was ~10 μL and contained 1 reaction buffer (Thermo-Fisher), 2.0 mM of each dNTP, 2.0 mM of each primer, 50 mM MgCl_2_, 10 ng of template genomic DNA, and 0.5 U Platinum Taq polymerase (Thermo-Fisher). The forward primers were labelled with FAM, NED, HEX or PET-TGT AAA ACG ACG GCC AGT-3’ (Schuelke, 2000). Reactions were performed under the following conditions with different annealing temperatures (Ta) for the microsatellites: 94 °C for 3 min; 35 cycles of 94 °C for 20 s; 50 °C (Che 34), 54 °C (Che18, Che 46, Che 59) or 55 °C (Cpy 58) for 45 s; and 72 °C for 1 min, finalizing with 72 °C for 10 min. PCR products were visualized under ultraviolet light in 2.5% agarose gel stained with 0.001% GelRed and later purified using isopropanol and 70% ethanol. The amplified DNA was denatured and size-fractionated using capillary electrophoresis on an Applied Biosystems Genetic Analyser (Thermo-Fisher) with a LIZ (500) molecular size standard (Thermo-Fisher).

### Genetic diversity analyses

The concatenated alignment of the plastid intergenic spacers was used in all analyses that were performed for each species separately. Haplotypes were identified using DNAsp 5.10.01 (Librado and Rozas, 2009) and haplotype evolutionary relationships were estimated using Network 5.0.0.1 (http://www.fluxus-engineering.com/sharenet.htm) via the median-joining method (Bandelt *et al.*, 1999). We performed summary statistics (haplotype and nucleotide diversities) and quantified the partitioning of genetic variation among different populations through AMOVA (Analysis of Molecular Variation - Excoffier *et al.*, 1992) with 1,000 permutations using *F*_ST_ (pairwise differences) in Arlequin 3.5.1.2 (Excoffier and Lischer, 2010). Additionally, we used *G*_ST_ standardized method (Hedrick, 2005) with 1,000 permutations to quantify the genetic diversity as performed in DNAsp. The Fu’s Fs (Fu, 1997) and Tajima’s D (Tajima, 1989) neutrality tests were also performed in Arlequin. The Bayesian skyline plot (BSP – Drummond *et al.*, 2005) analysis was performed using Beast 1.8 (Drummond *et al.*, 2012) for each species individually in order to evaluate the historical population size. For this analysis, a relaxed molecular clock model with a mean substitution rate of 2.810^-9^ per site per year (standard deviation 5.410^-11^) according to Lorenz-Lemke *et al.* (2010) and HKY nucleotide substitution model as estimated in JModelTest (Darriba *et al.*, 2012) were used as priors. Markov Chain Monte Carlo was performed for 100,000,000 steps, sampling every 10,000 steps. Tracer 1.6 (Rambaut *et al.*, 2013) was employed to compute the BSP and to inspect for convergence. We searched the literature on related species in order to compare haplotype and nucleotide diversity values with published data.

For microsatellite markers, the quality and size of the nuclear genotyped alleles were checked by using GeneMarker 2.2.0 (http://www.softgenetics.com/GeneMarker.php). The number of alleles per locus, allelic richness, *G*_ST,_ and fixation index (*F*) were estimated in FSTAT 2.9.3.2 (Goudet, 1995). Cervus 3.07 (Marshall *et al.*, 1998; Kalinowski *et al.*, 2007) was used to estimate the frequency of null alleles, the levels of observed (H_O_) and expected (H_E_) heterozygosity, and significant deviations from the Hardy–Weinberg equilibrium (HWE) were assessed after Bonferroni correction (p = 0.05).

The AMOVAs were conducted using 1,000 permutations among collection sites and *F*_ST_ (pairwise differences) using Arlequin, and we also performed a Principal Coordinates Analysis (PCoA) using GenAlEx 6.5 (Peakall and Smouse, 2012) based on a distance matrix (proportion of shared alleles) generated in MSA 4.05 (Dieringer and Schlötterer, 2003). In addition, the existence and number of genetic clusters for each species were inferred using STRUCTURE 2.3.4 (Pritchard *et al.*, 2000), and the best K value was estimated through ΔK (Evanno *et al.*, 2005) in CLUMPAK ONLINE (http://clumpak.tau.ac.il/contact.html). STRUCTURE runs were performed using 10^6^ Markov Chain Monte Carlo repetitions after a 10^5^ burn-in period and ten iterations per K, evaluating different numbers of possible clusters per species (1-6 for *C. eglandulata*, 1-5 for *C. sendtneriana,* and *C. serrulata,* and 1-8 for *C. spathulata*) to cover the number of different collection sites. The resulting bar plot from the summarized iterations for the best K was generated using CLUMPAK ONLINE.

### Conservation status assessment

We used the International Union for the Conservation of Nature (IUCN, 2017) Criteria and the online tool GeoCAT (Bachman *et al.*, 2011) to estimate the conservation status of *C. sendtneriana* and review the current status of *C. eglandulata, C. serrulata,* and *C. spathulata*. The GeoCAT input file included coordinates retrieved from SpeciesLink (CRIA, 2017) and our databank. Regarding the SpeciesLink data, we removed coordinates associated with misidentifications as well as those with the same collection number. IUCN Criteria were applied considering simultaneously our population structure results and population size estimates based on the number of individuals collected per site during field expeditions.

## Results

### Plastid markers

**Figure 2:**
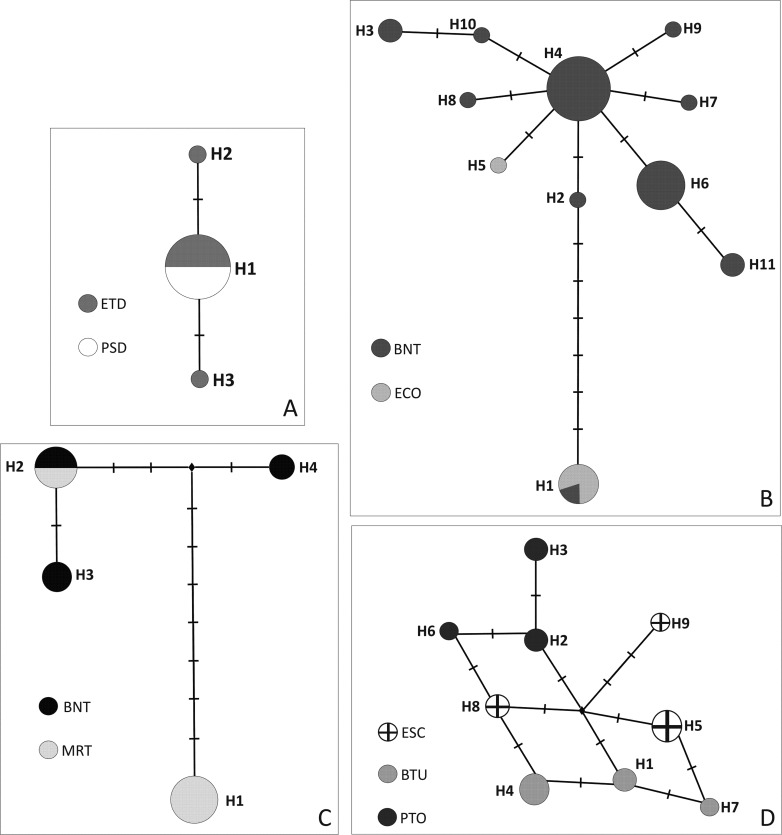
Evolutionary relationships of plastid haplotypes found in four *Calibrachoa* species (A) *C. eglandulata*; (B) *C. sendtneriana*; (C) *C. serrulata*; and (D) *C. spathulata* haplotypes. The circles represent haplotypes, and the diameter is proportional to the frequency across analyzed individuals per species.

The combined cpDNA (*trnG-trnS* and *psbA-trnH*) yielded a 1,181-bp sequence for each species in independent alignments, with two variable sites for *C. eglandulata*, 15 for *C. sendtneriana*, 11 for *C. serrulata,* and seven for *C. spathulata*. Nucleotide diversity values ranged from 0.02 to 0.43% (Table 2) and haplotype diversity ranged from 0.24 to 0.93% on average among species. *C. eglandulata* had the lowest values per species for both statistics (π = 0.02%; *h* = 0.24), with these results visible in the haplotype network (Figure 2A) where the haplotypes H2 and H3 present only one mutation in relation to H1. The highest value for nucleotide diversity per species was seen in *C. serrulata* (π = 0.43%), whereas the highest value of *h* was in *C. spathulata* (*h* = 0.93). Considering populations individually, PSD of *C. eglandulata* was monomorphic and MRT of *C. serrulata* presented the highest nucleotide diversity (π = 0.36), especially concerning the high number of mutations that separate H1 (exclusive to MRT) from the other haplotypes in this species. PTO population of *C. spathulata* showed the highest haplotype diversity (*h* = 0.80). The number of haplotypes among species ranged from three (*C. eglandulata*) to 11 (*C. sendtneriana*). In *C. eglandulata*, the two populations shared the most frequent haplotype, whereas two haplotypes were observed in only one individual each from the ETD population (Table 2); in *C. sendtneriana*, the most frequent haplotype was exclusive to the BNT population, and the BNT and ECO populations shared only one haplotype (Figure 2B). The individuals of two populations of *C. serrulata* shared only one haplotype (Figure 2C). All populations of *C. spathulata* presented exclusive haplotypes; there were three haplotypes in each population, and all of them were found in low frequencies (Table 2).

**Table 2:** Genetic variability and populations based on concatenated *trnH*-*psbA*/*trnS*-*trnG* for four *Calibrachoa* species.

Species/Populations	Haplotypes (AF)	π% (sd)	*H* (sd)	Fs	D
*C. eglandulata*	H1 (14), H2 (1), H3 (1)	0.02 ± 0.03	0.24 ± 0.14	-1.6	-1.2
ETD	H1, H2, H3	0.04 ± 0.04	0.42 ± 0.19		
PSD	H1	-	-		
*C. sendtneriana*	H1 (5), H2 (1), H3 (2), H4 (14), H5 (1), H6 (8), H7 (1), H8 (1), H9 (1), H10 (1), H11 (2)	0.23 ± 0.14	0.80 ± 0.05	-2.1	-0.8
BNT	H1, H2, H3, H4, H6, H7, H8, H9, H10, H11	0.13 ± 0.09	0.76 ± 0.06		
ECO	H1, H5	0.27 ± 0.20	0.40 ± 0.24		
*C. serrulata*	H1 (7), H2 (6), H3 (3), H4 (2)	0.43 ± 0.25	0.74 ± 0.06	-5.3	-2.2
BNT	H2, H3, H4	0.15 ± 0.11	0.75 ± 0.10		
MRT	H1, H2	0.36 ± 0.22	0.47 ± 0.13		
*C. spathulata*	H1 (2), H2 (2), H3 (2), H4 (3), H5 (3), H6 (1), H7 (1), H8 (2), H9 (1)	0.19 ± 0.12	0.93 ± 0.03	-3.4^#^	0.0
ESC	H5, H8, H9	0.15 ± 0.12	0.74 ± 0.16		
BTU	H1. H4, H7	0.08 ± 0.07	0.73 ± 0.16		
PTO	H2, H3, H6	0.08 ± 0.08	0.80 ± 0.16		

(AF) - absolute frequency; π - nucleotide diversity; *h* - haplotype diversity; sd - standard deviation; Fs - Fu’s Fs; D - Tajima’s D; ^#^ - significant value at p ≤ 0.02

According to the AMOVA results (Table 3), no population structure was detected in *C. eglandulata*; complete genetic diversity was attributed to the divergence among individuals within populations. For *C. sendtneriana*, the majority of diversity was found among populations (70%), whereas for *C. serrulata* and *C. spathulata* similar values (~50%) of genetic diversity were found among and within populations. In the case of *C*. *spathulata*, the genetic partitioning percentages found were related to the similar values obtained for π and *h* in each population (Table 2), with the resultant strong and varied population structure denoted in the haplotype network (Figure 2D).

**Table 3:** Population structure based on plastid and microsatellite markers through AMOVA, *F*_ST_, and *G*_ST_ analyses.

		Source of variation			
		Among Populations	Within Populations	*F*_ST_	*G*_ST_
cpDNA	*C. eglandulata*	0.0	100.0	-0.03	0.03
	*C. sendtneriana*	70.0	30.0	0.70	0.17
	*C. serrulata*	53.8	46.2	0.54	0.19
	*C. spathulata*	52.6	47.4	0.53	0.29
SSR	*C. eglandulata*	18.0	82.0	0.18	0.18
	*C. sendtneriana*	1.6	98.4	0.02	0.02
	*C. serrulata*	9.3	90.7	0.09	0.11
	*C. spathulata*	24.3	75.7	0.24	0.25

cpDNA – concatenated *trnH*-*psbA*/*trnS*-*trnG;* SSR – five microsatellite nuclear loci.

The *F*_ST_ values (Table 3) were higher than *G*_ST_‘s, which can be related to the mutation rate of the markers employed in our study. The presence of population structure for *C*. *spathulata* was evident in both methods (*F*_ST_ = 0.53 and *G*_ST_ = 0.289), as well as the lack of population structure in *C*. *eglandulata* (*F*_ST_ = -0.03 and *G*_ST_ = 0.029). In *C*. *serrulata*, the *F*_ST_ value was similar to *C*. *spathulata’s* but *G*_ST_ indicated a moderate population structure (*G*_ST_ = 0.189). For *C*. *sendtneriana, F*_ST_ pointed to strong population structure whereas *G*_ST_ indicated a moderate structure (*F*_ST_ = 0.70 and *G*_ST_ = 0.173, respectively). All values for neutrality tests, except for Fu’s Fs in *C. spathulata*, were non-significant (Table 2).

The demographic patterns of each species as assessed through the Bayesian skyline plot analysis (Figure S5) indicated stable population sizes for *C. eglandulata* (despite high standard deviations probably due to low genetic diversity) and *C. spathulata*. *C. serrulata* data suggested a population decrease in the last 100,000 years (also with high standard deviation values). For *C. sendtneriana*, the BSP indicated a population expansion in the last 100,000 years, which was compatible with the star-like shape of the haplotype network (Figure 2B). However, these results should be considered with caution due to the large credibility intervals associated with population genetic diversity estimates.

### Nuclear markers

The numbers of alleles per locus and per species ranged from three to 27 and the locus that presented the highest number of alleles was Che46 in all species. In general, the proportion of null alleles was low among loci and species (0.01% to 0.84%), and Che18 was the locus that showed the highest number of null alleles for all species. At least one locus per species deviated from HWE after Bonferroni correction (p = 0.05), indicating a heterozygotes deficit (Table S3).

The mean number of alleles per locus among the five microsatellite loci varied from 8.2 (*C. serrulata*) to 12.6 (*C. sendtneriana*) (Table 4). Allelic richness ranged from 7.7 (*C. eglandulata*) to 10.4 (*C. sendtneriana*). The fixation index (F) values were significant for all species and populations except PSD (*C. eglandulata*), BNT (*C. serrulata*), and PTO (*C. spathulata*).

**Table 4:** Genetic diversity and demographic indices for four *Calibrachoa* species based on nuclear microsatellites and populations.

Species/Population		N	A/L	AR	*H*_*O*_	*H*_*E*_	*F*
*C. eglandulata*		54	9.2	7.7	0.48	0.59	0.19^*^
	ETD	40	8.2	6.2	0.46	0.55	0.16^*^
	PSD	14	3.4	3.4	0.53	0.54	0.02
*C. sendtneriana*		54	12.6	10.4	0.60	0.76	0.21^*^
	BNT	31	9.8	9.2	0.60	0.77	0.23^*^
	ECO	23	9	8.8	0.59	0.73	0.19^*^
*C. serrulata*		30	8.2	7.8	0.45	0.64	0.31^*^
	BNT	10	5.2	4.9	0.50	0.59	0.16
	MRT	20	7	5.1	0.42	0.62	0.33^*^
*C. spathulata*		48	9.8	9.1	0.43	0.60	0.41^*^
	ESC	27	6.8	3.3	0.40	0.58	0.31^*^
	BTU	12	5	3.5	0.44	0.62	0.31^*^
	PTO	9	3.6	2.8	0.44	0.60	0.29

N - Number of individuals A/L - Alleles per *Locus*; AR - Allelic Richness; H_O_ - Observed Heterozygosity; H_E_ - Expected Heterozygosity; *F* – Fixation Index

* p < 0.05.

**Figure 3:**
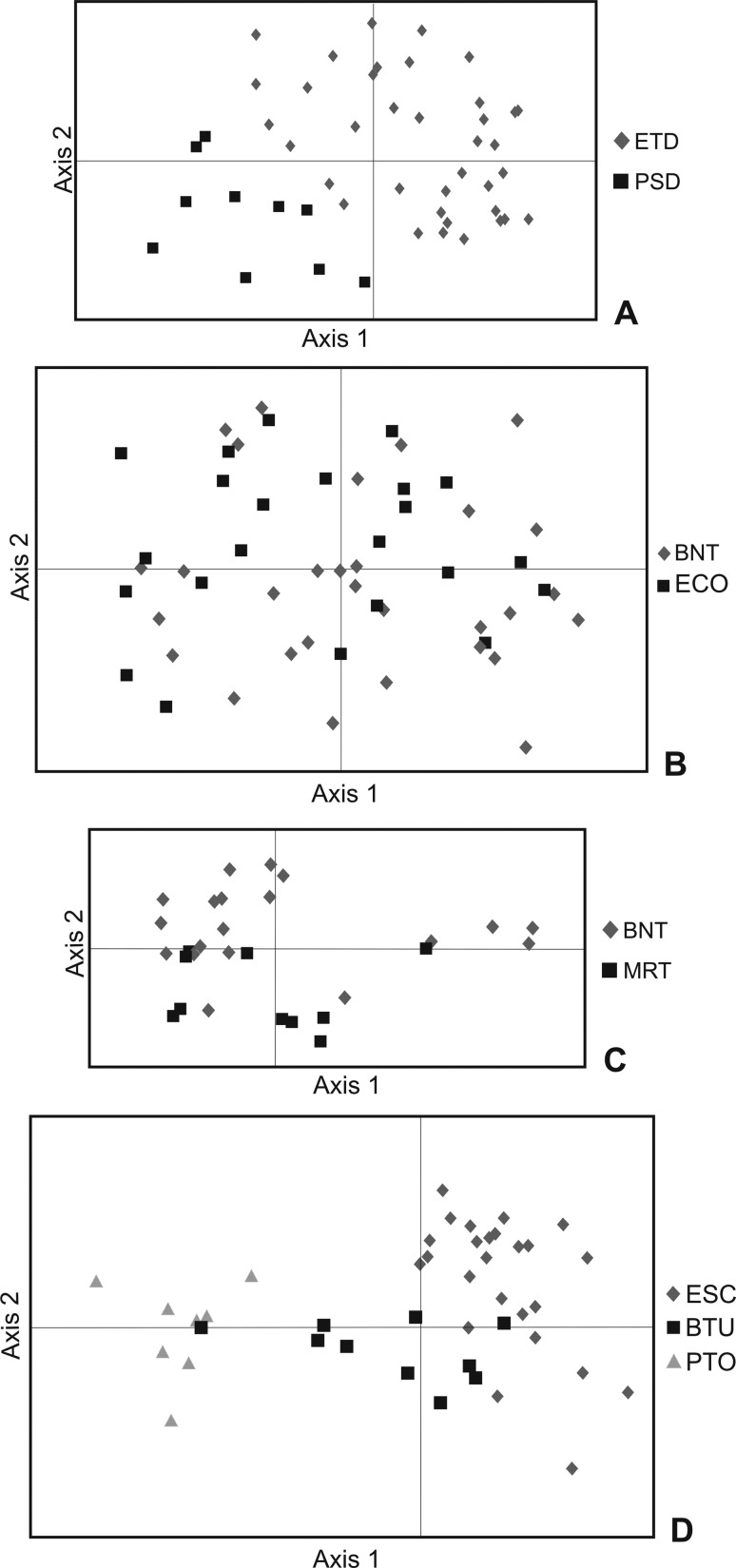
Ordination of individual microsatellite profiles of four *Calibrachoa* species in a principal coordinate analysis (PCoA) with the first two vectors per species: (A) *C. eglandulata*; (B) *C. sendtneriana*; (C) *C. serrulata*; and (D) *C. spathulata*. Individuals are labeled according to the legend.

Based on the five microsatellite loci (Table S3), the four *Calibrachoa* species presented higher genetic diversity within populations than among populations. The highest divergence among populations was observed in *C. spathulata* (24.3%), and the lowest value was seen in *C. sendtneriana* (1.6%), with this same pattern recovered in *F*_ST_ and *G*_ST_ estimates for both species (Table 3): *F*_ST_ = 0.24 and *G*_ST_ = 0.25 for the former, and *F*_ST_ = 0.02 and *G*_ST_ = 0.02 for the latter. *C. eglandulata* presented moderate values of *F*_ST_ and *G*_ST_, and for *C. serrulata* a slight indication of population structure is suggested, with *F*_ST_ < *G*_ST._ PCoA analysis (Figure 3A-D) detected a population structure in *C. eglandulata* and identified ETD and PSD as differentiated; in *C. spathulata* the individuals were grouped into their respective populations, and in *C. serrulata* some individuals from one population were positioned closer to individuals from another population than those from their own population. Absence of population structure was found for *C. sendtneriana*.

The genetic clustering based on STRUCTURE analysis and best K values (Figure S6) revealed just one genetic component for each *C. serrulata* and *C. sendtneriana*, suggesting no differentiation between populations in these species; there were three components in *C. eglandulata* and four in *C. spathulata*. For *C. eglandulata*, ETD individuals presented different proportions of each component, whereas individuals from PSD were more homogeneous, predominantly presenting the less frequent component seen in ETD. Individuals from the PTO population of *C. spathulata* were homogeneous and presented only one genetic component, whereas individuals from ESC and BTU displayed the four genetic components in different proportions.

### Conservation status assessment

Having increased the amount of information concerning the geographical distribution of these four species, we re-evaluated their conservation status according the IUCN (2017) criteria and suggest they can be updated as follow: the conservation status of *C. eglandulata* was unaltered, and the species remains categorized as Endangered [EN B1+2ab (ii, iii)]; item ii was added because the species presents a continued decline in its area of occupancy (AOO = 16 km^2^) due to advances in the construction of a roadway. Moreover, *C. eglandulata* inhabits a highly fragmented habitat with loss of quality and area and is found in only two locations.

*Calibrachoa sendtneriana* was categorized as Endangered (EN D) because fewer than 250 mature individuals were found. This estimate was based on the number of individuals sampled per collection site throughout ten years of field expeditions. Fragmentation of the species distribution was also considered.

The conservation status of *C. serrulata* was reviewed, and the species is now categorized as Vulnerable (VU D2) because of its restricted AOO = 12 km^2^ and the small number of locations where these individuals can be found.

The status of *C. spathulata* was also changed; the species is now categorized as Endangered [EN B1+2ab(iii)] due to the AOO = 44 km^2^ and the extent of occurrence (EOO) = ~1300 km^2^. The fragmentation of habitat and the formation of subpopulations along with habitat quality loss caused by urbanization and land usage contributed to the increased threat of species survival.

## Discussion

Rare species can be described as naturally rare or old rare species (Ciéslak *et al.*, 2015), and be associated with singular environments or geographical distribution. The BSHG occupies small and isolated areas (Behling, 2002), with grass vegetation covering the hilltops and *Araucaria* forest dominating the protected valleys (Safford, 1999). The relationship between grassland and forest during the Quaternary climate changes in this region is well documented through pollen records (Behling, 2002) and phylogeographic studies, especially in *Petunia* (Lorenz-Lemke *et al.*, 2010) and *Calibrachoa* (Fregonezi *et al.*, 2013).

Shifts in vegetation range, particularly the grassland expansion during the Pleistocene glacial periods, allowed the ancestral dispersion of *Petunia* (Reck-Kortmann *et al.*, 2014), whereas the geographical isolation caused by contraction of open fields during the interglacial periods drove diversification (Lorenz-Lemke *et al.*, 2010). Similar patterns have been suggested to explain the distribution of *Calibrachoa* highland species (Fregonezi *et al.*, 2012, 2013).

Despite the general consensus that species that are naturally rare present structured populations and low genetic diversity (Gitzendanner and Soltis, 2000), some rare species show high genetic diversity and little or no interpopulation differentiation (Turchetto *et al.*, 2016).

Here, we presented plastid and nuclear genetic data for four narrowly endemic, rare, and threatened *Calibrachoa* species. The data revealed different levels of genetic diversity and population structure between markers and across species. Population size and reduced distribution seem not to have influenced the genetic diversity for *C. serrulata*, which presented nucleotide diversity similar to species with a large geographic distribution such as *C. heterophylla* and *P. integrifolia* spp. *integrifolia* (Table S2). *C. serrulata* also showed high nuclear genetic diversity despite having the lowest value of allele richness among the four studied *Calibrachoa* species, which suggests that the high genetic diversity is not a consequence of ancestral polymorphisms (Jakob and Blattner, 2006) but rather is an inherent trait of this species.

Factors such as the type of pollination, mating system, seasonality of pollination at community level, and longevity of flowers may influence gene flow patterns, and the gene flow impacts directly on the genetic diversity and population structure (Barrett, 2010). Pollination in *C*. *serrulata* is performed by hummingbirds (Fregonezi *et al.*, 2012), which favors pollen exchange and outcrossing (Franceschinelli and Bawa, 2000) and long-distance pollen flow, as seen between BNT and MRT populations with the weak or absent population structure recovered in *F*_ST_ and *G*_ST_ for nuclear markers, PCoA and STRUCTURE analyses. As this is a perennial species, generation overlap could also influence the variability estimates. Despite that, this species presented high fixation index values, particularly in the MRT population. This can be attributed to biparental inbreeding, because the restricted seed dispersal (as seeds fall and germinate close to the mother plant; van der Pijl, 1982) causes intrapopulation spatial genetic structure, and near-neighbor individuals are probably genetically related individuals. Therefore, the high fixation index might reflect cohort mixing rather than an actual increased level of inbreeding. Combined with habitat loss, high levels of inbreeding constitute the main risk for *C. serrulata*.

The plastid genetic diversity indices in *C. sendtneriana* were low to moderate compared to other species (Table S2), whereas this species had the highest values of genetic diversity in nuclear markers among the four analyzed *Calibrachoa* species. Low diversity in plastid sequences could be a consequence of founder effects (Shirk *et al.*, 2014), small effective size, (Gibson *et al.*, 2008), or severe or continual bottlenecks (Castro *et al.*, 2015), while discrepancies between diversity as estimated through different genomes could be attributed to differences in coalescence time (Li *et al.*, 2002). The high genetic diversity observed in *C. sendtneriana* based on microsatellite loci might be explained by gene flow among populations (indirectly indicated by the *F*_ST_ and *G*_ST_ values; Heller and Siegismund, 2009) promoted by long-distance pollen flow (Ellstrand and Elam, 1993) as this species is bird-pollinated (Fregonezi *et al.*, 2013). Since this species occurs in sympatry with other *Calibrachoa* species, we cannot discard the possibility of interspecific gene flow, as the species in this genus preserve the intercrossing capacity (Watanabe *et al.*, 1997) at least in controlled conditions. Fragmented distribution, population size, and potential introgression coupled to habitat degradation could heighten the risk of *C. sendtneriana* extinction.

When we compared markers, *C. spathulata* showed high to moderate levels of population structure. The populations of this species are located far from each other, limiting the amount of interpopulation gene flow both by seed or pollen dispersion through barochory and bee-mediated. Moreover, this species presented the highest fixation index value, which could be attributed to biparental inbreeding. *C. spathulata*, especially the PTO population, had a high density of individuals per population that bloomed simultaneously, favoring an increase in pollinator visit rates (Kunin, 1997), but because of the high probability of neighboring individuals to be genetically related, pollen exchange in this case can occur between relatives or even parents and offspring since the species is perennial and overlapped generations can be observed in a single blooming season. The main risk factors for this species are habitat loss and fragmented distribution.

Among the studied *Calibrachoa* species, *C. eglandulata* presented the lowest diversity values for both plastid and nuclear genomes. Based on microsatellites, gene flow between populations is highly reduced or absent because of the geographical distance among populations and the pollinator behavior, since this species is pollinated by solitary bees that can fly only short distances (Stehmann and Semir, 2001). This isolated population structure and the low level of genetic diversity constitute the main threats to *C. eglandulata*.

The data generated in this study allow different scenarios to be depicted regarding genetic diversity, population size, and geographical range of each *Calibrachoa* species. *C. serrulata* can be considered a naturally rare species, like *Petunia secreta* (Turchetto *et al.*, 2016), with restricted geographic range (AOO = 12 km^2^; EOO = 0.993 km^2^) but high genetic diversity. Nuclear genetic diversity distributed throughout the two known populations makes both equally important reservoirs of variability, and therefore both should be protected in order to maintain the species’ adaptive potential (Binks *et al.*, 2015).

*C. sendtneriana* also displays little or no population differentiation based on nuclear markers, although BNT population harbors the majority of plastid haplotypes found in this species. Even though it has a larger geographic range (AOO = 36 km^2^; EOO = 282.9 km^2^) and the highest allelic richness, *C. sendtneriana* is more prone to habitat loss than *C. serrulata* because it occurs in areas partially converted into pasture and forest borders directly affected by human interferences, whereas *C. serrulata* grows vertically in the canyon walls and is theoretically more protected from habitat loss.

*C. spathulata* has the widest geographic range of the four endemic species analyzed here (AOO = 44 km^2^ and EOO = 1,264 km^2^) and presents the largest population size. Considering the strong population structure associated with exclusive haplotypes, conservation actions towards this species should aim to protect all known populations, especially considering that individuals of this species grow on roadsides and other highly urbanized areas. Based on these results and statements by Casacci *et al.* (2014), each population of *C. spathulata* could correspond to an evolutionary significant unit (ESU).

The habitat of *C. eglandulata* is highly fragmented with known populations located on roadsides. Because populations are small and isolated and present low genetic diversity, *ex situ* conservation may be necessary for this species.

The high fixation index values and heterozygote deficit in the four *Calibrachoa* species came as no surprise, because the population sizes are small and related individuals tend to grow next to each other due to limited seed dispersal in these species. Combined, these two conditions may be considered as an additional threat to species survival and conservation because the species would tend to lose genetic variability over time due to mating between relatives. We recommend monitoring these species in future years, not only through estimates of population sizes but also with special attention paid to their genetic diversity and potential habitat loss.

The importance of genetic diversity in the maintenance of biological diversity and in evolutionary processes is well established, especially considering the predictions of climate change (Barros *et al.*, 2015). Conservation strategies based on genetic analysis, however, are still limited in the BSHG (Overbeck *et al.*, 2007). The species studied herein have low to high levels of plastid genetic diversity compared to related species. We also observed low to high levels of population structure as a result of restricted pollen and seed dispersal based on nuclear markers. Plants presenting biparental inbreeding are more likely to suffer from a loss of alleles, since aggregated populations or even cohorts in a small area can be eliminated by human activity or natural phenomena (Ellstrand, 2014).

In conclusion, the genetic diversity and population structure found in these four rare and narrowly endemic *Calibrachoa* species may be attributed to historical events, mating systems, and pollinators, whereas the fragmented range and small population sizes are a consequence of habitat loss due to human activities. To ensure the species’ survival, actions such as local protection and *ex situ* conservation would be necessary.

## Supplementary material

The following online material is available for this article:

Table S1 GenBank accession numbers and plastid haplotypes.

Table S2 Plastid genetic diversity of *Petunia* and *Calibrachoa* species.

Table S3 Genetic diversity per locus per species for four *Calibrachoa* species

Figure S1  Calibrachoa eglandulata.

Figure S2 *Calibrachoa sendtneriana*.

Figure S3  Calibrachoa serrulata.

Figure S4  Calibrachoa spathulata.

Figure S5 Bayesian skyline plot showing the fluctuations in effective population size (Ne) over time per *Calibrachoa* species.

Figure S6 Population structure and evolutionary relationships of individuals of four *Calibrachoa* species based on five microsatellite loci;
