# Supplementary material for: Genetic diversity and population structure of naturally rare Calibrachoa species with small distribution in southern Brazil
Source: Genet Mol Biol. 2019 Mar 11;42(1):108–19. doi: 10.1590/1678-4685-GMB-2017-0314 (PMC6428134; doi:10.1590/1678-4685-GMB-2017-0314)
Supplement: Supplementary file 1 [file 1415-4757-GMB-1678-4685-GMB-2017-0314-20190214-suppl4.pdf]

Supplementary Material to "Genetic diversity and population structure of naturally rare *Calibrachoa* species with small distribution in southern Brazil"

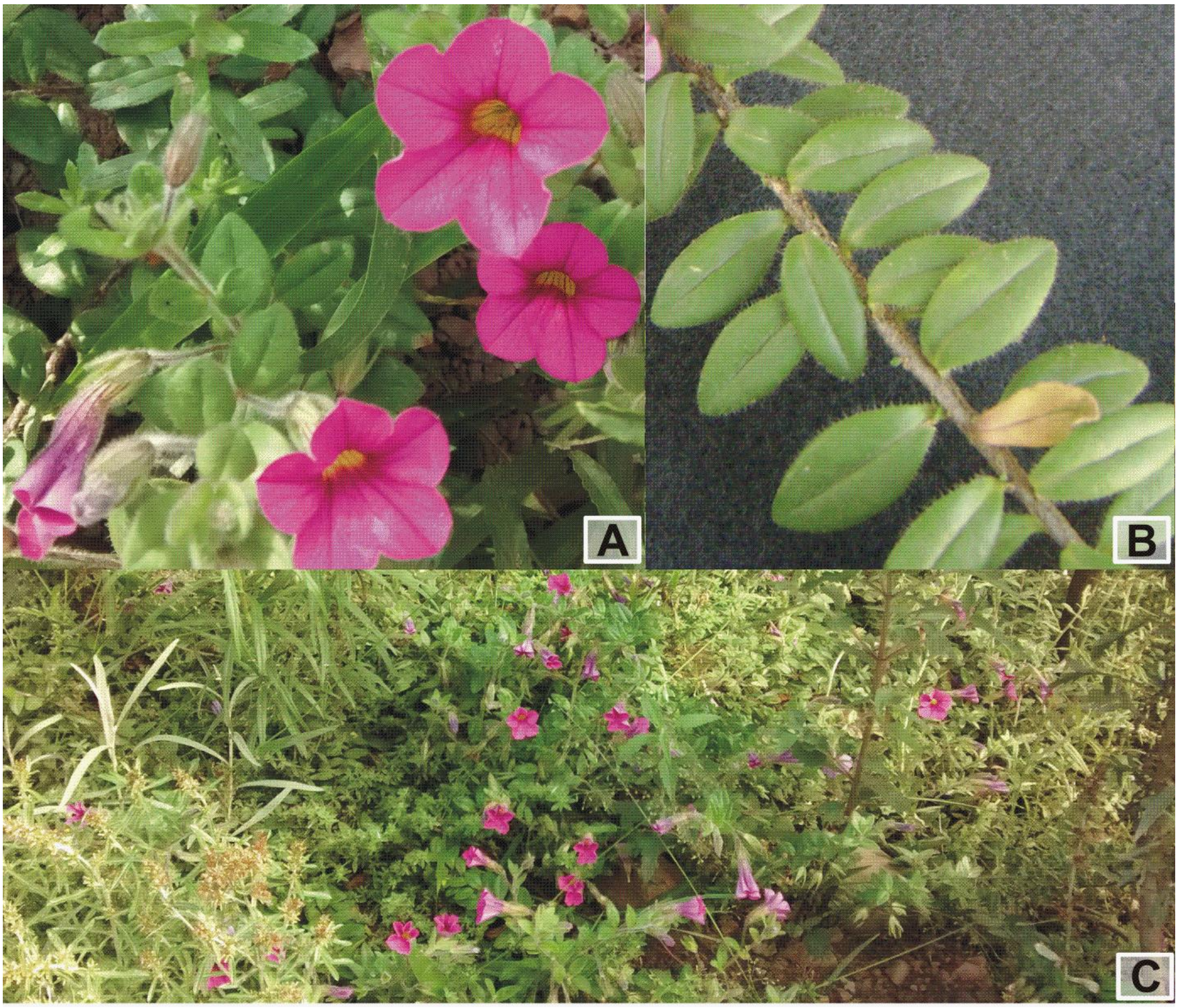

Figure S1 - *Calibrachoa eglandulata*: A) flowers; B) leaves; C) habitat.
