# Supplementary material for: Genetic diversity and population structure of naturally rare Calibrachoa species with small distribution in southern Brazil
Source: Genet Mol Biol. 2019 Mar 11;42(1):108–19. doi: 10.1590/1678-4685-GMB-2017-0314 (PMC6428134; doi:10.1590/1678-4685-GMB-2017-0314)
Supplement: Supplementary file 5 [file 1415-4757-GMB-1678-4685-GMB-2017-0314-20190214-suppl1.pdf]

Supplementary Material to "Genetic diversity and population structure of naturally rare *Calibrachoa* species with small distribution in southern Brazil"

Table S1 - GenBank numbers and haplotype information of four *Calibrachoa* species.

Species	Individual code	Population code	Haplotype code	GenBank Accession number	
				trnH-psbA	*trnS-trnG*
C. eglandulata	egla08	EDT	H1A	KY630252	KY630335
	egla11	PSD	H1A	MF431228	KY630336
	egla12	PSD	H1A	MF431229	KY630337
	egla13	PSD	H1A	MF431230	KY630338
	egla14	PSD	H1A	MF431231	KY630339
	egla15	PSD	H1A	MF431232	KY630340
	egla17	PSD	H1A	MF431233	KY630341
	egla19	PSD	H1A	MF431234	KY630342
	egla21	EDT	H1A	KY630253	KY630344
	egla24	EDT	H1A	MF431235	KY630347
	egla26	EDT	H1A	MF431236	KY630348
	egla28	EDT	H1A	MF431237	KY630350
	egla29	EDT	H2A	MF431238	KY630351
	egla32	EDT	H1A	KY630255	KY630353
	egla35	EDT	H1A	KY630256	KY630356
	egla39	EDT	H3A	KY630257	KY630360
C. sendtneriana	send01	ECO	H1B	KY630264	KY630364
	send04	ECO	H1B	KY630265	KY630365
	send06	BNT	H2B	KY630267	KY630366
	send07	BNT	H3B	KY630268	KY630367
	send08	ECO	H1B	KY630269	KY630368
	send09	ECO	H1B	KY630270	KY630369
	send10	ECO	H1B	KY630271	KY630370

Species	Individual code	Population code	Haplotype code	GenBank Accession number	
				trnH-psbA	*trnS-trnG*
	send11	BNT	H4B	KY630272	KY630371
	send12	BNT	H4B	KY630273	KY630372
	send16	BNT	H8B	KY630274	KY630376
	send17	BNT	H4B	KY630275	KY630377
	send18	BNT	H4B	KY630276	KY630378
	send19	BNT	H4B	KY630277	KY630379
	send23	BNT	H4B	KY630278	KY630381
	send24	BNT	H4B	KY630279	KY630382
	send26	BNT	H4B	KY630280	KY630384
	send31	BNT	H4B	KY630281	KY630386
	send32	BNT	H9B	KY630282	KY630387
	send33	BNT	H4B	KY630283	KY630388
	send34	BNT	H4B	KY630284	KY630389
	send35	BNT	H4B	KY630285	KY630390
	send40	BNT	H6B	KY630286	KY630394
	send42	BNT	H6B	KY630287	KY630395
	send46	BNT	H10B	KY630288	KY630396
	send47	BNT	H11B	KY630289	KY630397
	send48	BNT	H3B	KY630290	KY630398
	send50	BNT	H11B	KY630291	KY630399
	send52	BNT	H6B	KY630292	KY630400
	send53	BNT	H6B	KY630293	KY630401
	send54	BNT	H6B	KY630294	KY630402
	send55	BNT	H6B	KY630295	KY630403
	send111	ECO	H5B	KY630302	KY630404
	send112	BNT	H6B	KY630303	KY630405
	send114	BNT	H6B	KY630304	KY630406
	send117	BNT	H7B	KY630305	KY630407
	send119	BNT	H4B	KY630306	KY630408

Species	Individual code	Population code	Haplotype code	GenBank Accession number	
				trnH-psbA	*trnS-trnG*
	send120	BNT	H4B	KY630307	KY630409
C. serrulata	serr01	MRT	H1C	KY630308	KY630410
	serr02	MRT	H1C	KY630309	KY630411
	serr03	MRT	H1C	KY630310	KY630412
	serr11	MRT	H2C	KY630311	KY630420
	serr13	BNT	H2C	MF431216	KY630422
	serr14	BNT	H3C	KY630312	KY630423
	serr15	BNT	H3C	MF431217	KY630424
	serr16	BNT	H2C	KY630313	KY630425
	serr17	BNT	H3C	KY630314	KY630426
	serr18	BNT	H4C	KY630315	KY630427
	serr19	BNT	H4C	KY630316	KY630428
	serr20	MRT	H1C	KY630317	KY630429
	serr21	MRT	H1C	KY630318	KY630430
	serr23	MRT	H1C	KY630319	MF431239
	serr26	MRT	H1C	KY630320	KY630431
	serr27	MRT	H2C	KY630321	KY630432
	serr28	MRT	H2C	KY630322	KY630433
	serr29	BNT	H2C	KY630323	KY630434
C. spathulata	spat03	BTU	H4D	KY630325	MF431251
	spat06	BTU	H4D	KY630326	KY630436
	spat14	BTU	H7D	KY630327	KY630437
	spat23	BTU	H4D	KY630328	MF431239
	spat30	ESC	H8D	KY630329	MF431240
	spat32	ESC	H8D	KY630330	KY630438
	spat35	ESC	H9D	KY630331	KY630439
	spat87	ESC	H5D	MF431218	MF431241
	spat93	ESC	H5D	MF431219	MF431242
	spat105	ESC	H5D	MF431220	MF431243

Species	Individual code	Population code	Haplotype code	GenBank Accession number	
				trnH-psbA	*trnS-trnG*
	spat116	BTU	H1D	MF431221	MF431244
	spat118	BTU	H1D	MF431222	MF431245
	spat130	PTO	H6D	KY630333	KY630440
	spat131	PTO	H2D	MF431224	MF431247
	spat132	PTO	H3D	MF431225	MF431248
	spat140	PTO	H2D	MF431226	MF431249
	spat144	PTO	H3D	MF431227	MF431250
