# Supplementary material for: Genetic diversity and population structure of naturally rare Calibrachoa species with small distribution in southern Brazil
Source: Genet Mol Biol. 2019 Mar 11;42(1):108–19. doi: 10.1590/1678-4685-GMB-2017-0314 (PMC6428134; doi:10.1590/1678-4685-GMB-2017-0314)
Supplement: Supplementary file 6 [file 1415-4757-GMB-1678-4685-GMB-2017-0314-20190214-suppl8.pdf]

Supplementary Material to "Genetic diversity and population structure of naturally rare *Calibrachoa* species with small distribution in southern Brazil"

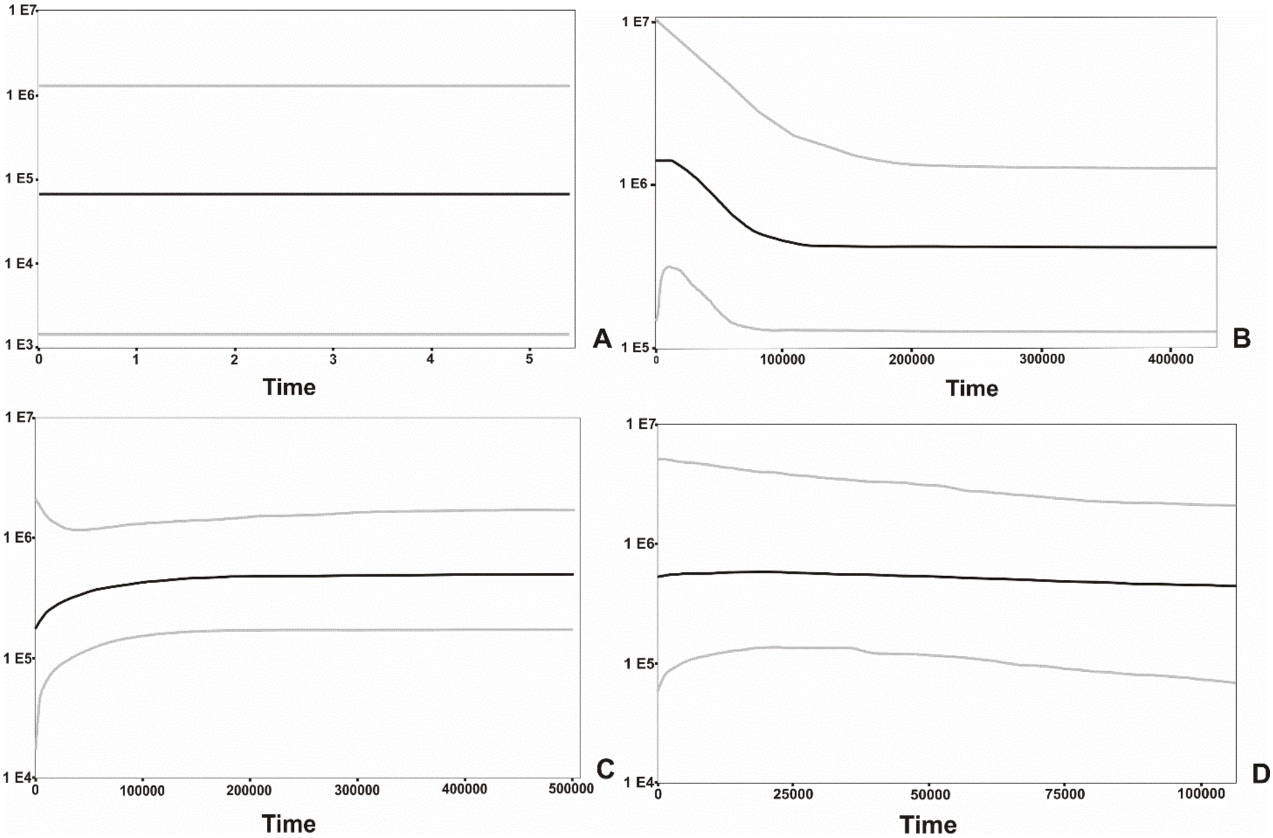

Figure S5 - Bayesian skyline plot showing the fluctuations in effective population size (Ne) over time per *Calibrachoa* species: (A) *C. eglandulata*; (B) *C. sendtneriana*; (C) *C. serrulata*; and (D) *C. spathulata*. The dark line indicates the median estimate, and the grey area represents the 95% highest posterior density interval.
