# Supplementary material for: Genetic diversity and population structure of naturally rare Calibrachoa species with small distribution in southern Brazil
Source: Genet Mol Biol. 2019 Mar 11;42(1):108–19. doi: 10.1590/1678-4685-GMB-2017-0314 (PMC6428134; doi:10.1590/1678-4685-GMB-2017-0314)
Supplement: Supplementary file 7 [file 1415-4757-GMB-1678-4685-GMB-2017-0314-20190214-suppl3.pdf]

Supplementary Material to "Genetic diversity and population structure of naturally rare *Calibrachoa* species with small distribution in southern Brazil"

Table S3 - Genetic diversity per locus for four *Calibrachoa* species considering five nuclear microsatellite loci.

Species	Locus	Length (bp)	NA	H_O	H_E	Null (%)
C. eglandulata	Che18	124-175	7	0.30*	0.64	0.36
	Che34	217-233	6	0.60	0.68	0.07
	Che46	195-293	24	0.91	0.93	0.01
	Che59	91-115	6	0.15	0.25	0.24
	Cpy58	191-195	3	0.44	0.48	0.02
C. sendtneriana	Che18	115-199	18	0.28*	0.90	0.53
	Che34	219-229	6	0.64	0.52	0.14
	Che46	201-291	27	0.77*	0.96	0.10
	Che59	91-117	7	0.54*	0.65	0.12
	Cpy58	181-199	5	0.76	0.76	0.01
C. serrulata	Che18	130-166	5	0.17*	0.62	0.57
	Che34	219-227	4	0.46	0.45	0.01
	Che46	225-301	20	0.73	0.93	0.11
	Che59	109-115	4	0.47	0.56	0.11
	Cpy58	159-201	8	0.39	0.65	0.24
C. spathulata	Che18	124-184	9	0.06*	0.74	0.84
	Che34	215-231	6	0.24*	0.59	0.42
	Che46	203-285	23	0.76	0.93	0.09
	Che59	89-115	6	0.52	0.62	0.07
	Cpy58	193-253	5	0.40	0.45	0.03

NA - Number of Alleles; H_O - Observed Heterozygosity; H_E - Expected Heterozygosity; Null - Null Alleles; *HWE deviation, significance after Bonferroni correction ($p > 0.05$).
