# Supplementary material for: Genetic diversity and population structure of naturally rare Calibrachoa species with small distribution in southern Brazil
Source: Genet Mol Biol. 2019 Mar 11;42(1):108–19. doi: 10.1590/1678-4685-GMB-2017-0314 (PMC6428134; doi:10.1590/1678-4685-GMB-2017-0314)
Supplement: Supplementary file 8 [file 1415-4757-GMB-1678-4685-GMB-2017-0314-20190214-suppl9.pdf]

Supplementary Material to "Genetic diversity and population structure of naturally rare *Calibrachoa* species with small distribution in southern Brazil"

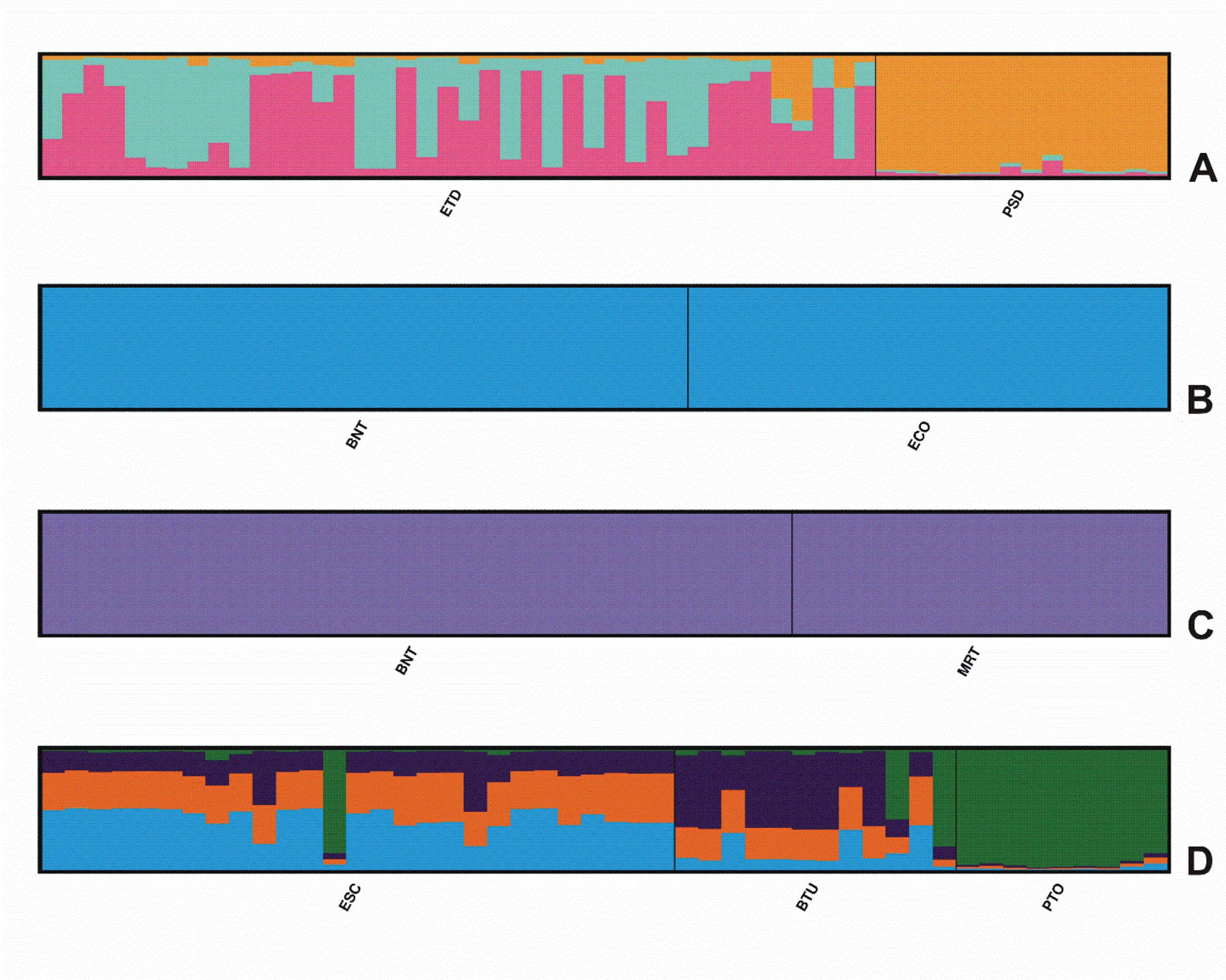

Figure S6 - Population structure and evolutionary relationships of individuals of four *Calibrachoa* species based on five microsatellite loci as observed through clustering analysis in STRUCTURE. Different colors indicate groups according to the best K (Evanno *et al.*, 2005) per species, and vertical lines correspond to individuals. Population codes follow Table 1 in the main text. (A) *C. eglandulata*; (B) *C. sendtneriana*; (C) *C. serrulata*; and (D) *C. spathulata.*
