# Supplementary material for: Genetic diversity and population structure of naturally rare Calibrachoa species with small distribution in southern Brazil
Source: Genet Mol Biol. 2019 Mar 11;42(1):108–19. doi: 10.1590/1678-4685-GMB-2017-0314 (PMC6428134; doi:10.1590/1678-4685-GMB-2017-0314)
Supplement: Supplementary file 9 [file 1415-4757-GMB-1678-4685-GMB-2017-0314-20190214-suppl2.pdf]

Supplementary Material to "Genetic diversity and population structure of naturally rare *Calibrachoa* species with small distribution in southern Brazil"

Table S2 - Nucleotide diversity values retrieved from the literature for *Calibrachoa* and *Petunia* species obtained for concatenated alignment of the intergenic spacers *trnH-psbA*/*trnS-trnG*.

Species	N	H	π % (sd)	Study*
Calibrachoa eglandulata	16	3	0.02 (0.03)	This study
Calibrachoa sendtneriana	37	11	0.23 (0.14)	This study
Calibrachoa serrulata	18	4	0.43 (0.25)	This study
Calibrachoa spathulata	17	9	0.19 (0.12)	This study
Calibrachoa heterophylla	247	27	0.41 (0.22)	Mäder *et al.* 2013
Petunia integrifolia spp. *depauperata*	289	25	0.13 (0.09)	Ramos-Fregonezi *et al*. 2015
Petunia integrifolia spp *integrifolia*	24	9	0.40 (0.2)	Longo *et al.* 2014
Petunia riograndensis	7	6	0.20 (0.2)	Longo *et al.* 2014
Petunia inflata	34	12	0.26 (0.16)	Segatto *et al*. 2014b
Petunia interior	89	23	0.34 (0.19)	Segatto *et al*. 2014b
Petunia hybrida	22	3	0.35 (0.20)	Segatto *et al*. 2014b
Petunia exserta	322	6	0.10 (0.1)	Segatto *et al*. 2014a
Petunia secreta	65	9	0.17 (0.11)	Turchetto *et al*. 2016
Petunia axillaris complex	614	35	0.22 (0.13)	Turchetto *et al*. 2014

N - Number of individuals; H - Number of haplotypes; π - nucleotide diversity; sd - standard deviation; *original sources

References

Longo D, Lorenz-Lemke AP, Mäder G, Bonatto SL and Freitas LB (2014) Phylogeography of the *Petunia integrifolia* complex in southern Brazil. Bot J Linn Soc 174:199-213.

Mäder G, Fregonezi JN, Lorenz-Lemke AP, Bonatto SL and Freitas LB (2013) Geological and climatic changes in quaternary shaped the evolutionary history of *Calibrachoa heterophylla*, an endemic South-Atlantic species of petunia. BMC Evol Biol 13:178.

Ramos-Fregonezi AMC, Fregonezi JN, Cybis G, Fagundes NJR, Bonatto SL and Freitas LB (2015). Were sea level changes during the Pleistocene in the South Atlantic Coastal Plain a driver of speciation in *Petunia* (Solanaceae)? BMC Evol Biol 15:92.

Segatto ALA, Cazé ALR, Turchetto C, Klahre U, Kuhlemeier C, Bonatto SL and Freitas LB (2014a) Nuclear and plastid markers reveal the persistence of genetic identity: A new perspective on the evolutionary history of *Petunia exserta*. Mol Phylogenet Evol 70:504-512.

Segatto ALA, Ramos-Fregonezi AMC, Bonatto SL and Freitas LB (2014b) Molecular insights into the purple-flowered ancestral of garden petunias. Am J Bot 101:119-127.

Turchetto C, Fagundes NJR, Segatto ALA, Kuhlemeier C, Neffa VGS, Speranza PR, Bonatto SL and Freitas LB (2014) Diversification in the South American Pampas: The genetic and morphological variation of the widespread *Petunia axillaris* complex (Solanaceae). Mol Ecol 23:374-389.

Turchetto C, Segatto ALA, Mäder G, Rodrigues DM, Bonatto SL and Freitas LB (2016) High levels of genetic diversity and population structure in an endemic and rare species: implications for conservation. AoB Plants 8:plw002.
